# NPC1 regulates ER contacts with endocytic organelles to mediate cholesterol egress

**DOI:** 10.1038/s41467-019-12152-2

**Published:** 2019-09-19

**Authors:** D. Höglinger, T. Burgoyne, E. Sanchez-Heras, P. Hartwig, A. Colaco, J. Newton, C. E. Futter, S. Spiegel, F. M. Platt, E. R Eden

**Affiliations:** 1 Department of Pharmacology, University of Oxford, Oxford, UK; 2 Heidelberg University Biochemistry Center (BZH), Heidelberg, Germany; 3 UCL Institute of Ophthalmology, London, UK; 4 Paediatric Respiratory Department, Royal Brompton Hospital, London, UK; 5 Center for Autophagy, Recycling and Disease, Danish Cancer Society Research Center, Copenhagen, Denmark; 6 Department of Biochemistry and Molecular Biology, Virginia Commonwealth University School of Medicine, Richmond, VA 23298 USA

**Keywords:** Lysosomes, Lysosomes

## Abstract

Transport of dietary cholesterol from endocytic organelles to the endoplasmic reticulum (ER) is essential for cholesterol homoeostasis, but the mechanism and regulation of this transport remains poorly defined. Membrane contact sites (MCS), microdomains of close membrane apposition, are gaining attention as important platforms for non-vesicular, inter-organellar communication. Here we investigate the impact of ER-endocytic organelle MCS on cholesterol transport. We report a role for Niemann-Pick type C protein 1 (NPC1) in tethering ER-endocytic organelle MCS where it interacts with the ER-localised sterol transport protein Gramd1b to regulate cholesterol egress. We show that artificially tethering MCS rescues the cholesterol accumulation that characterises NPC1-deficient cells, consistent with direct lysosome to ER cholesterol transport across MCS. Finally, we identify an expanded population of lysosome-mitochondria MCS in cells depleted of NPC1 or Gramd1b that is dependent on the late endosomal sterol-binding protein STARD3, likely underlying the mitochondrial cholesterol accumulation in NPC1-deficient cells.

## Abstract (web summary)

Though endocytosed dietary cholesterol is transferred to the endoplasmic reticulum (ER), how this is regulated is unclear. Here, the authors report a role for Niemann-Pick Type C Protein 1 (NPC1) in tethering endocytic organelles to the ER, which may contribute to interorganelle cholesterol transport.

## Introduction

Cholesterol is an essential lipid that maintains membrane integrity and serves as a precursor of several classes of signalling molecules. However, cholesterol accumulation in the endocytic pathway is associated with neurological diseases^1^, such as Niemann-Pick type C (NPC), a progressive childhood neurodegenerative disease characterised on a cellular level by the accumulation of cholesterol and multiple sphingolipid species in lysosomes^2^. Mammalian cells have therefore evolved sophisticated systems to regulate cholesterol homoeostasis. Upon endocytosis, dietary low-density-lipoprotein (LDL)-derived cholesterol is transported to the ER, where sterol levels are sensed and endogenous cholesterol biosynthesis is downregulated accordingly^3^. How cholesterol reaches the ER from endocytic organelles remains unclear but likely involves several parallel pathways^4^. Some LDL-cholesterol is thought to traffic via the plasma membrane, thereby safeguarding plasma membrane cholesterol supply prior to downregulation of cholesterol production at the ER^4^. However, a substantial body of evidence supports direct transport of approximately 30% of LDL-cholesterol from endosomes to the ER^5,6^. The oxysterol-binding protein homologue ORP1L has recently been implicated in cholesterol transport to the ER^7,8^. ORP1L is a late endosomal protein that binds ER-localised vesicle associated membrane protein (VAMP)-associated proteins (VAPs) through a conserved FFAT motif^9^ and this interaction is important for ORP1L’s role in cholesterol transport^10^. Studies using the virally encoded protein RIDα are consistent with a role for ORP1L in LDL-cholesterol transport to the regulatory pool in the ER^11^. Interestingly, under conditions of sterol depletion, the ORP1L-VAP interaction is implicated in cholesterol transport in the opposite direction, from ER to endosomes, to support the formation of intraluminal vesicles (ILV) within the endosome^12^, suggesting a role for these membrane contact site (MCS) proteins in bidirectional cholesterol transport. Another endosomal sterol-binding protein, STARD3, also interacts with VAPs at the ER to promote ER to endosome cholesterol transport and ILV formation^13^.

NPC disease is caused by mutations in genes encoding lysosomal proteins NPC1 (95% of cases) or NPC2 (5% of cases). NPC1 is a large transmembrane protein, which localises to the limiting membrane of late endocytic organelles. NPC2 is small soluble luminal protein that shuttles cholesterol from intra-lysosomal vesicle membranes to the N-terminal domain of NPC1^14^. However, the function of NPC1 is less well understood and it has previously been proposed to be a cholesterol transporter or a cholesterol regulated transporter of multiple substrates. A previous report indicates that NPC1 interacts with the ER-resident oxysterol-binding protein ORP5 and that both NPC1 and ORP5 are required for LDL-cholesterol egress from endocytic organelles to the ER^15^. This finding adds to the body of evidence suggesting a role for ER-endosome MCS as conduits for direct LDL-cholesterol transport to the ER^16–18^.

Here we show that the cholesterol environment of the endocytic pathway influences the interactions made by lysosomal sterol-binding proteins to define the MCS populations formed with either ER or mitochondria. We find that NPC1 regulates ER contact sites with late endocytic organelles where it interacts with the ER-localised sterol transport protein Gramd1b. Critically, expansion of the MCS is sufficient to rescue the lysosomal accumulation of LDL-derived cholesterol in the absence of NPC1 and mediate its transport to the ER.

## Results

### NPC1 tethers ER contact sites with late endocytic organelles

Using NPC1-deficient cells as models of defective cholesterol transport, we investigated the contribution of MCS to the egress of LDL-cholesterol from endocytic organelles using two complementary techniques: Live-cell fluorescence microscopy, enabling rapid screening of cellular organelles in their native environment, and electron microscopy (EM) where the higher resolution allows the contact itself to be visualised.

**Fig. 1:**
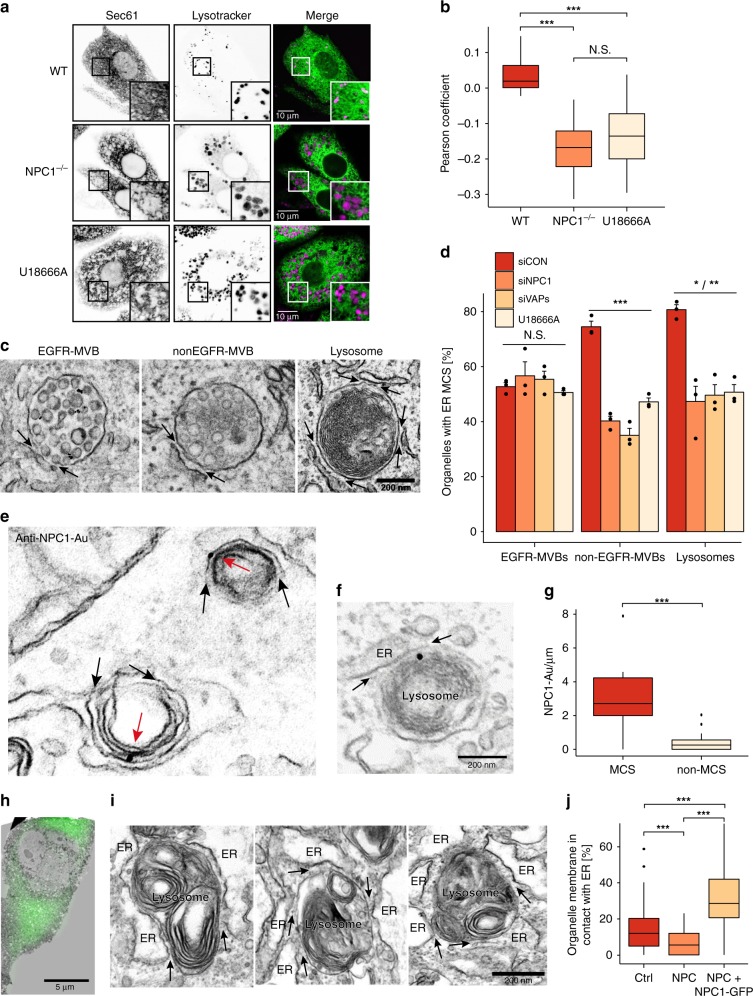
NPC1 tethers ER contact sites with late endocytic organelles. **a** Live-cell confocal microscopy images of ER and late endosomes/lysosomes. WT Chinese hamster ovary (CHO) cells, NPC1^−/−^ CHO cells and WT CHO cells treated with U18666A (2 µg/ml for 72 h) were transfected with Sec61-GFP for 24 h and incubated with Lysotracker Red (75 nM) for 15 min before imaging. Scale bar, 10 µm. **b** Quantification of ER and lysosomal co-localization. The Pearson correlation coefficient between Sec61-GFP and Lysotracker channels was extracted for each cell (WT: *n* = 24 cells, NPC1^−/−^: *n* = 30 cells, U18666A: *n* = 27 cells) and presented as boxplots. Centre lines show medians, box limits indicate first (Q1) and third quartiles (Q3), whiskers extend to a maximum distance of 1.5*IQR (interquartile range) from Q1 and Q3, respectively or to the most extreme datapoint within that range. Welch two sample t-tests were performed between all conditions (WT-NPC: ***P = 2.8 × 10^−15^, WT-U18666A: ***P = 8.6 × 10^−12^, NPC-U18666A: N.S. *P* = 0.185). **c** Representative electron micrographs showing distinct MCS populations. HeLa cells were stimulated with EGF for 25 min in the presence of anti-EGFR antibody coupled to 10 nm gold prior to preparation for EM. Arrows indicate MCSs between the ER and endocytic organelles. Scale bar, 200 nm. **d** Endocytic organelle populations in **c** were scored according to the presence of MCS with the ER and the percentage of organelles with an ER MCS quantified. Data shown is the mean of three independent experiments + SEM. Welch two sample t-tests were performed between siCON and treatment conditions for each vesicle population (EGFR-MVBs: N.S. *P* = 0.524, *P* = 0.470, *P* = 0.278, nonEGFR-MVBs: ***P = 0.00025, *P* = 0.00034, *P* = 0.00071, Lysosomes: */**P = 0.01859, *P* = 0.00622, *P* = 0.00164). **e** Representative electron micrograph of NPC-patient fibroblasts transfected with NPC1-GFP and stained for NPC1 using pre-embedding labelling. NPC1-GFP (red arrows) is visible at MCSs (black arrows) between the ER and lysosomes. Scale bar, 200 nm. **f** Representative electron micrograph showing endogenous NPC1 staining at ER-lysosome MCSs (black arrows) using pre-embedding labelling in HeLa cells. **g** Quantification of NPC1 labelling on late endosome/lysosome limiting membrane that is (MCS) or is not (nonMCS) in contact with the ER (*n* = 16 late endosomes/lysosomes). **h** NPC1-GFP was expressed in NPC-patient fibroblasts cultured on gridded dishes and imaged by light microscopy prior to preparation for EM. EM images of individual cells, identified using grid coordinates, were montaged and the fluorescent image overlaid in order to distinguish between expressing and non-expressing cells. An expressing cell is shown. Scale bar, 5 μm. **i** Representative electron micrographs of endocytic organelles in NPC1gfp-expressing cells showing extended MCSs (arrows). Scale bar, 200 nm. **j** The length of MCSs was measured in non-transfected control fibroblasts (Ctrl) and transfected NPC-patient fibroblasts not expressing (NPC) or expressing NPC1-GFP (+NPC1gfp) and expressed as a percentage of the total endocytic organelle limiting membrane. *n* = 62 organelles (Ctrl), 60 organelles (NPC) and 84 organelles (+NPC1gfp). Welch two sample *t*-tests were performed between all conditions (Ctrl/NPC: ***P = 7.4 × 10^−5^, Ctrl/NPCgfp: ***P = 1.8 × 10^−9^, NPC/NPCgfp: ***P < 2.2 × 10^−16^)

We first examined the association between late endosomes/lysosomes and the ER in control and *Npc1*^*−/−*^ Chinese hamster ovary (CHO) cells by fluorescence microscopy. Using Sec61-GFP and LysoTracker to visualise ER and lysosomes respectively, we found significantly reduced association between the two organelles in *Npc1*^*−/−*^ compared to control cells as quantified using Pearson’s correlation coefficient (Fig. 1a, b). A similar reduction in co-localization was achieved by acute pharmacological NPC1 inhibition using U18666A, which binds the sterol-sensing domain of NPC1 and induces cholesterol accumulation^19^ (Fig. 1a, b).

We previously found that different populations of endocytic organelles are tethered to the ER by distinct protein complexes. For example, epidermal growth factor receptor (EGFR)-containing multi-vesicular endosomes/bodies (MVBs) are tethered by Annexin-A1 and its calcium-dependent ligand S100A11, while other non-EGFR populations are tethered by VAPs^12^. We therefore examined the relative impact of NPC1 downregulation (Supplementary Fig. 1a) on different ER-endosome MCS populations by EM. HeLa cells were stimulated with EGF in the presence of an antibody to EGFR’s extracellular domain coupled to gold, to distinguish EGFR-containing endosomes from other endocytic organelle populations (Fig. 1c). While Annexin-A1-regulated ER contacts with EGFR-containing MVBs were unchanged by NPC1 depletion, ER contacts with EGFR-negative MVBs (nonEGFR-MVBs) and lysosomes were reduced by approximately 50%, mirroring our previously reported effect of VAP depletion^12^ (Fig. 1d). Treatment with U18666A also specifically reduced ER contacts with nonEGFR-MVBs and lysosomes (Fig. 1d). Consistent with a role for NPC1 in regulating MCS, ER-nonEGFR-MVB and ER-lysosome MCS populations were also significantly reduced in NPC1 patient fibroblasts compared to controls (Supplementary Fig. 1b). In contrast, these changes in MCS were not observed in NPC2 patient fibroblasts, likely because NPC2 is a luminal protein and cannot therefore function in tethering MCS (Supplementary Fig. 1b).

To further explore the role of NPC1 in stabilising MCS, we investigated its subcellular localisation by immuno-EM. As expected, NPC1-GFP (Fig. 1e) or endogenous NPC1 (Fig. 1f and Supplementary Fig. 1c and d) localised predominantly to the limiting membrane of late endosomes and lysosomes, including at ER-endocytic organelle MCS. Furthermore, NPC1 appears enriched at the MCS, with a six-fold increase in the number of gold particles per micron of the limiting membrane in contact with the ER compared to regions not associated with the ER (Fig. 1g). We next measured the effect of NPC1 overexpression on the extent of ER-endocytic organelle MCS in NPC-patient fibroblasts using correlative light and EM to identify NPC1-GFP-expressing cells (Fig. 1h). ER contacts with endocytic organelles were significantly extended in cells over-expressing NPC1-GFP (Fig. 1i, j).

Collectively, the reduction of MCS in the absence of NPC1, the presence of NPC1 at MCS and the expansion of MCS on NPC1 overexpression, indicate a role for NPC1 in tethering MCS between late endocytic organelles and the ER.

### NPC1 interacts with an ER-localised protein, Gramd1b at MCS

**Fig. 2:**
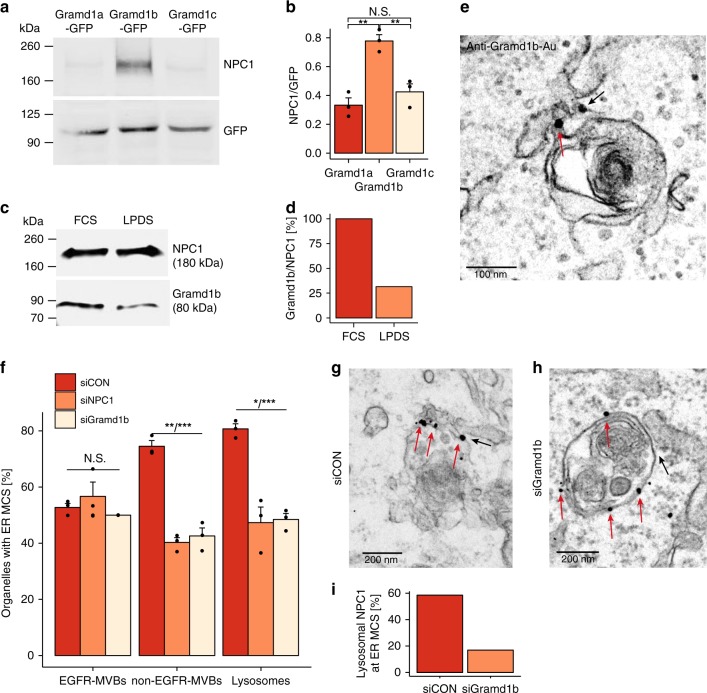
NPC1 interacts with the ER-localised sterol transport protein Gramd1b at MCSs. **a** GFP immunoprecipitates from HeLa cells transfected with Gramd1a-c GFP plasmids were immunoblotted with anti-NPC1 and GFP antibodies. **b** Quantification of the ratio of NPC to GFP present in **a**, represented as a mean of three independent experiments. Welch two sample *t*-tests were performed between all conditions (Gramd1a/Gramd1b: **P = 0.00275, Gramd1b/Gramd1c: **P = 0.00836, Gramd1a/Gramd1c: N.S. *P* = 0.2835). **c** NPC1 immunoprecipitates from HeLa cells cultured in medium containing 10% FCS or LPDS for 18 h were immunoblotted with anti-Gramd1b antibody. **d** Quantification of the amount of Gramd1b as a percentage of NPC1 present in c, represented as the mean of two experiments. **e** Representative electron micrograph showing endogenous Gramd1b staining at ER-lysosome MCSs (black arrows) using pre-embedding labelling in HeLa cells. Scale bar, 100 nm. **f** Endocytic organelle populations in HeLa cells treated with control, NPC1 or Gramd1b siRNA were scored according to the presence of MCS with the ER. The percentage of organelles with an ER MCS was quantified and represented as the mean of three independent experiments + SEM. Welch two sample *t*-tests were performed between siCON and treatment conditions for each vesicle population (EGFR-MVBs: N.S. *P* = 0.5244, *P* = 0.197, nonEGFR-MVBs: ***P = 0.00025, **P = 0.00127, Lysosomes: *P = 0.01859, ***P = 0.00038). **g**, **h** Representative electron micrographs showing endogenous NPC1 staining (red arrows) at an ER-lysosome MCS (black arrows) using pre-embedding labelling in HeLa cells transfected with a non-targeting control siRNA (**g**) or with siRNA targeting Gramd1b (**h**). Scale bar, 200 nm. **i** Quantification of NPC1 labelling on late endosome/lysosome limiting membrane that is (MCS) or is not (nonMCS) in contact with the ER in cells prepared in panels **g** and **h** (*n* = 50 late endosomes/lysosomes per condition)

Having established a role for NPC1 in MCS formation, we next sought to characterise its interactions at the contact site. A split reporter reassembly assay^20^ in yeast, using *Ncr1* (yeast NPC1) as bait, identified Lipid transfer protein Anchored at Membrane contact sites-4 (LAM4) as an interacting protein. LAMs are a family of lipid transporters that contain a GRAM domain in the pleckstrin homology superfamily and a StART (Steroidogenic Acute Regulatory protein-related lipid Transfer)-like domain, both of which are also present in three human proteins (GramD1a-c)^21^. Immunoprecipitation (IP) of GFP-Gramd1a-c proteins revealed a specific interaction between NPC1 and Gramd1b (Fig. 2a, b) that could also be detected by IP of endogenous proteins (Fig. 2c). This interaction is influenced by the sterol environment, as there was a reduction of Gramd1b coimmunoprecipitated with NPC1 from cells cultured in lipoprotein deficient serum (LPDS) compared with those cultured in the presence of full serum (Fig. 2c, d). The expression of both proteins appeared unaffected by the sterol environment (Supplementary Fig. 2a). NPC1 has previously been shown to interact with ORP5 when both proteins are overexpressed^15^. We were unable to detect an interaction between endogenous NPC1 and ORP5 under either sterol condition, but ORP5 expression is prohibitively low for reliable IPs of endogenous protein.

LAM4 has been shown to localise to the ER in yeast^21^. Live cell imaging of Gramd1b-GFP confirmed that Gramd1b is also an ER-resident protein (Supplementary Fig. 2b). Moreover, we found that, like NPC1, endogenous Gramd1b localises to lysosome-ER contact sites (Fig. 2e), including those positive for NPC1 (Supplementary Fig. 2c), consistent with these two proteins interacting at MCS. To explore the importance of this interaction in tethering the contacts, we quantified MCS in cells depleted of Gramd1b. Gramd1b depletion (Supplementary Fig. 2d) also reduced ER contacts with nonEGFR-MVBs and lysosomes to a similar extent as seen in NPC1-deficient cells (Fig. 2f). To further probe the importance of the Gramd1b interaction in NPC1’s role at MCS, we examined the effect of Gramd1b depletion on NPC1 localisation at the ER-lysosome interface. The enrichment of endogenous NPC1 label on late endosomes/lysosomes at sites of ER contact in control cells (Figs 1g, 2g, i) was lost on depletion of Gramd1b (Fig. 2h, i, Supplementary fig. 2e and f). This can in part be explained by the reduction in MCS in Gramd1b-deficient cells (Fig. 2f), but in late endocytic organelles with an ER contact, NPC1 label did not decorate the MCS as seen in controls (Fig. 2h, i). Taken together, these findings demonstrate a sterol-dependent interaction between sterol-binding proteins Gramd1b and NPC1 at the ER-lysosome interface to stabilise the interorganellar association.

### NPC1-regulated MCS mediate cholesterol transport to the ER

In addition to increasing MCS in NPC-patient fibroblasts, NPC1-GFP expression also rescued the cholesterol accumulation phenotype in the same cells (Supplementary Fig. 3a–d), suggesting that NPC1-dependent MCS might act as conduits for the transport of LDL-cholesterol to the ER. Therefore, we next examined the intracellular localization of an exogenously added “click”-cholesterol probe (pacChol) that combines a photoreactive group for UV crosslinking and an alkyne group for conjugation to an azide^22^. BSA-complexed pacChol was taken up by the endolysosomal pathway as indicated by vesicular staining in WT and NPC1^−/−^ cells after a 20 min pulse, which showed co-localization with the late endosomal marker LAMP1 (Supplementary Fig. 3e and f) at this early time-point. Longer chase times of 60 min led to nearly complete clearance of pacChol from lysosomes in WT cells, now giving rise to a reticular staining, whereas NPC1^−/−^ cells still retained pacChol in their lysosomes (Supplementary Fig. 3e and f, quantified in Supplementary Fig. 3g). These data substantiate a transport block at late-endosomal / lysosomal stages in NPC1-deficient cells. Since we have shown that NPC1 is required for MCS formation (Fig. 1d), this finding suggests that NPC1-regulated MCS might mediate cholesterol egress from the endocytic pathway.

**Fig. 3:**
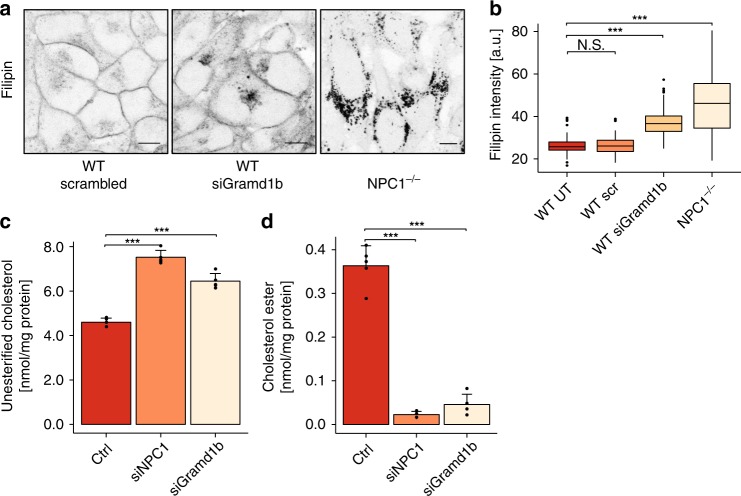
NPC1-regulated MCSs mediate cholesterol transport from late endocytic organelles to the ER. **a** Confocal microscopy images of WT HeLa cells transfected with control (scrambled) or Gramd1b siRNA as well as NPC1^−/−^ HeLa cells stained with 50 µg/mL Filipin. Scale bars,10 µm. **b** Quantification of Filipin intensity in cells treated as described in **a**. Boxplots show medians as centre lines, box limits indicate first (Q1) and third quartiles (Q3), whiskers are at a maximum distance of 1.5*IQR (interquartile range) from Q1 and Q3, respectively or to the most extreme datapoint within that range. Outliers are shown. Welch two sample t-tests were performed between untreated WT condition and treatment conditions: WT-scrambled (*n* = 95 cells), WT-siGramd1b (*n* = 101 cells), NPC1^−/−^ (*n* = 43 cells). N.S. *P* = 0.6216, ***P < 2.2 × 10^−16^, ***P = 4.9 × 10^−11^. **c** Quantification of unesterified cholesterol in HeLa cells treated with non-targeting control (ctrl), NPC1 and Gramd1b siRNAs. Cholesterol was measured using the Amplex Red Cholesterol Assay Kit and values are presented as mean of five independent experiments + SD. Welch two sample t-tests were performed between all conditions. ctrl/siNPC1 ***P = 0.00063, ctrl/siGramd1b **P = 0.00389, siNPC1/siGramd1b **P = 0.0085. **d** Quantification of cholesterol esters in HeLa cells treated with control, NPC1 and Gramd1b siRNAs. Cholesterol esters were measured using the Amplex Red Cholesterol Assay Kit with addition of 0.2 U/mL cholesterol esterase. Data are represented as mean of five independent experiments + SD. Welch two sample *t*-tests were performed between all conditions. ctrl/siNPC1 ***P = 0.00012, ctrl/siGramd1b ***P = 0.00033, siNPC1/siGramd1b N.S. *P* = 0.6758

We therefore examined the role of the NPC1-Gramd1b tethering complex in cholesterol egress. Filipin staining revealed an intracellular accumulation of cholesterol in Gramd1b-depleted cells, although to a lesser extent than in the absence of NPC1 (Fig. 3a, b). Filipin-stained cholesterol accumulated in a LAMP1-positive compartment in cells depleted of Gramd1b or NPC1 (Supplementary Fig. 3h), consistent with lysosomal accumulation. Since Filipin staining only yields qualitative information, we also sought to quantify intracellular cholesterol biochemically, using the AmplexRed cholesterol assay. Cells depleted of Gramd1b show elevated free cholesterol compared to control cells, but consistent with the Filipin staining, the elevation is weaker than in cells depleted of NPC1 (Fig. 3c). Gramd1b has recently been shown to also function in lipid transport at ER-plasma membrane MCS^23^. Since disruption of the lipid distribution at the plasma membrane can affect endocytosis^24^, we examined LDL uptake in Gramd1b-depleted cells. However, uptake of fluorescent-LDL was unchanged in Gramd1b-depleted cells compared with controls (Supplementary Fig. 3i). We also measured cholesterol ester levels in these cells as a measure of transport to the ER since esterification of excess cholesterol is catalysed by an ER-resident enzyme, Acyl-CoA:cholesterol acyltransferase (ACAT). Levels of cholesterol esters were significantly reduced in both NPC1 and Gramd1b-depleted cells (Fig. 3d), demonstrating impaired cholesterol transport to the ER when lysosome-ER association is reduced (Fig. 2f).

### MCS expansion restores cholesterol egress in NPC1^−/−^ cells

**Fig. 4:**
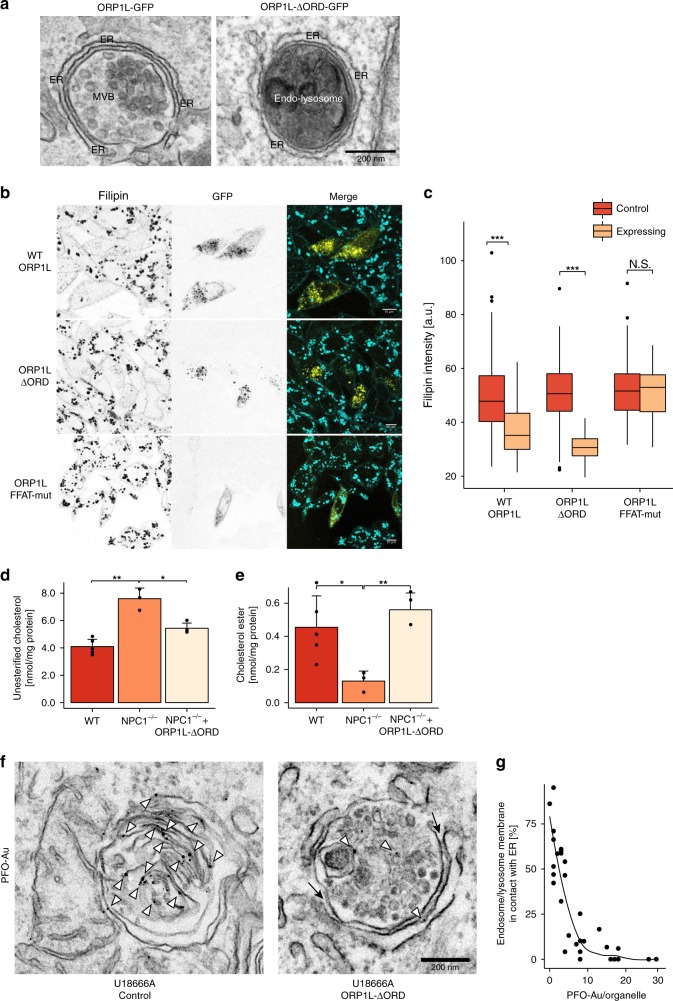
Expansion of lysosome-ER contact sites restores cholesterol egress in NPC1-deficient cells. **a** Electron micrographs showing extended MCSs in HeLa cells transfected with wtORP1L or ORP1L-ΔORD GFP constructs. Scale bar, 200 nm. **b** Confocal microscopy images of CHO NPC1^−/−^ cells transfected with ORP1L-GFP, ORP1L-deltaORD-GFP and ORP1L-FFATmut-GFP for 24 h, fixed and stained with 50 µg/ml Filipin (GFP, yellow; Filipin, cyan). Scale bar, 10 µm. **c** Quantification of intracellular Filipin staining in cells treated as described in **b**. Centre lines show medians, box limits indicate first (Q1) and third quartiles (Q3), whiskers extend to a maximum distance of 1.5*IQR (interquartile range) from Q1 and Q3, respectively or to the most extreme datapoint within that range. Welch two sample *t*-tests were performed between control and expressing cells for each condition (ORP1L: *n* = 203 cells (Control), *n* = 39 cells (Expressing), ***P = 9.1 × 10^−10^, ORP1L-deltaORD: *n* = 204 cells (Control), *n* = 42 cells (Expressing), ***P < 2.2 × 10^−16^, ORP1L-FFATmut-GFP: *n* = 202 cells (control), *n* = 33 cells (Expressing) N.S. *P* = 0.713,). **d** Quantification of unesterified cholesterol in WT and NPC1^−/−^ HeLa cells as well as in NPC1^−/−^ HeLa cells transfected with ORP1L-ΔORD-GFP, with expressing cells sorted by FACS. Cholesterol was measured using the Amplex Red Cholesterol Assay Kit and values are presented as mean of 5 (WT), 3 (NPC1) or 4 (NPC1 + ORP1L) independent experiments + SD. Welch two sample *t*-tests were performed: WT/NPC1^−/−^ **P = 0.00959, N*P*C1^−/−^/NPC1^−/−^ + ORP1L-ΔORD *P = 0.04161. **e** Quantification of cholesterol esters in WT and NPC1^−/−^ HeLa cells as well as in NPC1^−/−^ HeLa cells transfected with ORP1L-ΔORD-GFP and sorted by FACS. Cholesterol esters were measured using the Amplex Red Cholesterol Assay Kit with addition of 0.2 U/mL cholesterol esterase. Data are represented as mean of 5 (WT), 3 (NPC1) or 4 (NPC1 + ORP1L) independent experiments + SD with asterisks indicating statistical significance as determined by Welch two sample t-tests. WT/NPC1^−/−^ *P = 0.01547, N*P*C1^−/−^/NPC1^−/−^ + ORP1L-ΔORD **P = 0.00104. **f** HeLa cells transfected with ORP1L-deltaORD were treated with U18666A (2 µg/ml for 18 h) and labelled with PFO prior to preparation for EM. Representative electron micrographs show a lysosome with little/no contact with the ER (likely not expressing ORP1L-deltaORD) staining strongly for PFO and a lysosome with an extended contact with the ER with greatly reduced PFO labelling. Scale bar, 200 nm. **g** The percentage of endocytic organelle membrane in contact with the ER in **f** was measured and plotted against the number of PFO-gold particles (cholesterol label)/endocytic organelle (*n* = 30 organelles)

To further probe the role of MCS in the transfer of LDL-cholesterol to the ER, we examined the effect of MCS expansion on cholesterol transport. Overexpression of the MCS protein ORP1L-GFP dramatically increased ER-lysosome contacts (Fig. 4a) and also resulted in drastic reduction of accumulated cholesterol in NPC1-null cells, as shown by Filipin staining (Fig. 4b, c). However, the oxysterol binding-protein-related domain (ORD) of ORP1L is known to actively participate in cholesterol egress^7,8^. To determine whether ORP1L’s effect is due to its cholesterol transporting or its tethering properties, we made use of an ORP1L-delta-ORD mutant (ORP1L-ΔORD) which is known to act as a constitutively active tether between late endosomes and the ER without transporting cholesterol itself^10,12,18^. Remarkably, NPC1^−/−^ cells expressing ORP1L-ΔORD-GFP also showed reduced Filipin staining (Fig. 4b, c), even though LDL uptake was unaffected (Supplementary Fig. 4a). This reduction in free cholesterol can be attributed to increased ER-lysosome contacts (Fig. 4a) since in contrast, Filipin staining was not reduced in cells expressing a FFAT motif mutant ORP1L that is unable to tether to the ER^10^ (Fig. 4b, c).

We also examined the transport of cholesterol to the ER at these ORP1L-ΔORD-tethered contact sites. To this end, NPC1-deficient cells expressing ORP1L-ΔORD-GFP were FACS-sorted for GFP. Overexpression of ORP1L-ΔORD-GFP significantly decreased the accumulation of free cholesterol and markedly increased cholesterol esters compared to non-expressing cells (Fig. 4d, e), indicative of increased transport to the ER. Thus, the tethering function of ORP1L is critical for cholesterol egress from late endosomes/lysosomes in NPC1-deficient cells.

To directly correlate the degree of MCS expansion with cholesterol egress from endocytic organelles, cholesterol staining in U18666A-treated Hela cells transfected with ORP1L-ΔORD was examined by immuno-EM. Pre-embedding labelling with the cholesterol-binding toxin perfringolysin-O (PFO)^12,25^ was reduced in endocytic organelles with ORP1L-ΔORD-induced extended ER MCS (Fig. 4f), revealing an inverse relationship between the amount of PFO-stained cholesterol in the endocytic organelle and the proportion of its membrane in contact with the ER (Fig. 4g). Since pre-embedding labelling requires cell permeabilisation, and potential loss of membrane cholesterol, we also used cryo-immunoEM, where ultrathin sections are labelled without permeabilisation. ORP1L-ΔORD-GFP expression reduced PFO-labelled cholesterol in endocytic organelles of U18666A-treated cells by 68% (Supplementary Fig. 4b and c).

Taken together, these data indicate that NPC1-regulated MCS mediate direct transport of cholesterol from late endocytic organelles to the ER.

### Lysosome-mitochondria MCS are increased in NPC1^−/−^ cells

**Fig. 5:**
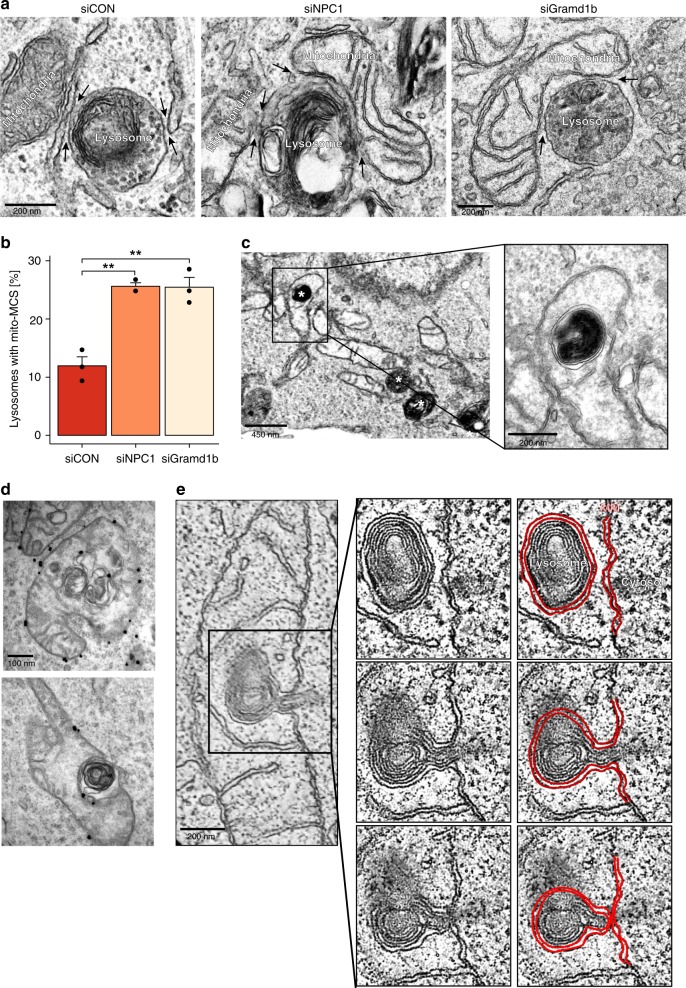
Increased lysosome-mitochondria contact sites in NPC1-deficient cells. **a** Electron micrographs of HeLa cells treated with non-targeting control siRNA (siCON), NPC1-siRNA (siNPC1) or with Gramd1b-siRNA (siGramd1b) showing lysosome MCSs (black arrows) with ER in control or mitochondria in NPC1 or Gramd1b-depleted cells. Scale bar, 200 nm. **b** The percentage of endocytic organelles with a mitochondria MCS from **a** were quantified and expressed as the mean of three independent experiments + SEM. Welch two sample t-tests were performed between siCON and treatment conditions (siNPC1:**P = 0.00615, siGramd1b:**P = 0.00412). **c** Electron micrograph of HeLa cells pulsed with HRP for 2 h prior to treatment with U18666A (2 µg/mL for 18 h). Asterisks indicate HRP-containing organelles, identified by the electron-dense reaction product. Scale bar, 450 nm and 200 nm in boxed enlargement **d** Representative electron micrographs of HeLa cells treated with U18666A (2 µg/mL for 18 h) and stained for endogenous Tom20 using pre-embedding labelling. Tom20-stained OMM appears to surround the lysosome. Scale bar, 100 nm. **e** Slices from a tomographic reconstruction from HeLa cells treated with U18666A (2 µg/mL for 18 h). Red lines, mitochondrial membrane. Scale bar, 200 nm

EM analysis of cells depleted of NPC1 or Gramd1b (Fig. 5a), or of NPC1-inhibited cells (Supplementary Fig. 5a), where lysosome-ER MCS are reduced (Figs. 1d and 2f) uncovered a reciprocal expansion of a population of MCS between lysosomes and mitochondria. Closer examination revealed similar contacts with visible tethers in control cells (Supplementary Fig. 5b) but these contacts were twice as abundant in cells depleted of NPC1 or Gramd1b (Fig. 5b) or in U18666A-treated cells (Supplementary Fig. 5c). Lysosome-like structures occasionally appeared partially (Supplementary Fig. 5d) or completely (Supplementary Fig. 5e) engulfed by mitochondria in NPC1-deficient or U18666A-treated cells. To confirm that these structures are lysosomes, the endocytic pathway was loaded with horseradish peroxidase (HRP) prior to treatment with U18666A. Electron dense HRP reaction product was clearly visible in late endocytic organelles, including those apparently inside mitochondria (Fig. 5c).

This appearance of lysosomes within the mitochondria could be suggestive of inter-organelle fusion. However, in the example shown in Fig. 5c, the lysosome is surrounded by a double membrane, characteristic of the mitochondrial membrane, which would signify a lack of fusion. ImmunoEM showed that the membrane around the lysosome was positive for the outer mitochondria membrane (OMM) protein Tom20 (Fig. 5d). Moreover, electron tomography showed the lysosome pushing into, but not through the OMM (Supplementary video). Successive tomographic slices through the mitochondria show the mitochondrial membrane surrounding rather than fusing with, the associated lysosome (Fig. 5e). These data show that in the absence of NPC1, lysosomes form extensive and stable MCS with mitochondria, but without membrane fusion between the two organelles.

### Increase in lysosome-mitochondria MCS is STARD3-dependent

**Fig. 6:**
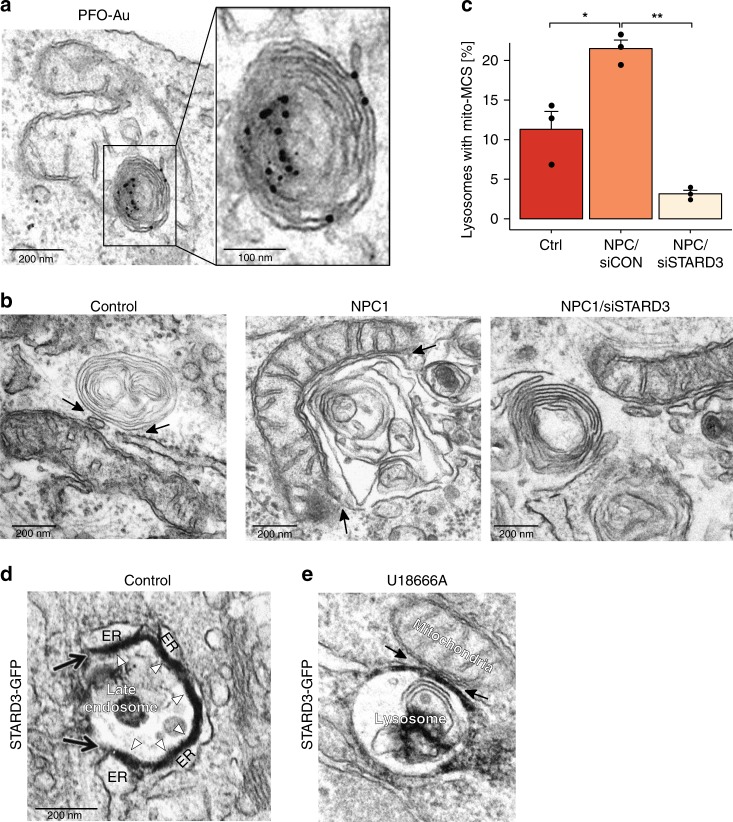
STARD3-dependent association of mitochondria with cholesterol-rich lysosomes with reduced ER contact. **a** Electron micrograph of HeLa cells treated with U18666A (2 µg/mL for 18 h) showing apparent engulfment of a lysosome by a mitochondria. Cholesterol was stained with PFO using pre-embedding labelling. “Engulfed” lysosomes are rich in PFO-stained cholesterol. Scale bar, 200 nm and 100 nm in boxed enlargement. **b** Representative electron micrographs showing lysosome contact sites (arrows) with ER or mitochondria in fibroblasts derived from healthy donors (control), fibroblasts from NPC1-patients (NPC1) or NPC1 patient fibroblasts treated with siSTARD3 (NPC1/siSTARD3). Scale bar, 200 nm. **c** The percentage of lysosomes with a mitochondria MCS from **b** are presented as mean from 3 independent experiments + SEM. **d**, **e** STARD3-GFP was co-expressed with APEX2-GBP in **d** control HeLa cells, or **e** HeLa cells treated with U18666A (2 µg/mL for 18 h) prior to preparation for EM. Electron dense APEX reaction product (STARD3-GFP) localizes to extended ER contacts with late endocytic organelles in control cells (**d**), but to lysosome-mitochondria MCS on NPC1 inhibition (**e**). Arrows, MCSs. Scale bar, 200 nm

PFO-staining revealed that mitochondria-surrounded lysosomes are laden with cholesterol (Fig. 6a), suggesting that high lysosome cholesterol content might promote mitochondrial association. Elevated mitochondrial cholesterol was previously reported in NPC cells^26^, prompting us to speculate that lysosome-mitochondria MCS could provide an alternative route for cholesterol egress in NPC cells. The late endosome/lysosome sterol transfer protein STARD3 has been implicated in LDL-cholesterol transport to mitochondria^26^. We found that depletion of STARD3 (Supplementary Fig. 6a) in NPC-patient fibroblasts drastically reduced lysosome-mitochondria contacts to a level well below that in control cells (Fig. 6b, c), indicating an essential role for STARD3 in the formation of these MCS. STARD3 overexpression is known to promote ER-endocytic organelle MCS^27^, but has not previously been implicated in lysosome-mitochondria contacts. On co-expression of STARD3-GFP with APEX-GBP (a GFP-binding peptide with a modified soybean ascorbate peroxidase (APEX) tag^28^), to allow STARD3 to be localised by EM, we found ER-endocytic organelle MCS to be greatly extended in control cells, consistent with previous reports^27^, with STARD3-GFP clearly visible as an electron-dense APEX reaction product at the MCS (Fig. 6d and Supplementary Fig. 6b). Surprisingly, when cells were treated with the NPC1 inhibitor U18666A, ER contact with late endocytic organelles was not increased and endosomal STARD3-GFP re-localised to the expanded lysosome-mitochondria interface (Fig. 6e and Supplementary Fig. 6c).

Thus, we have revealed a dual functionality for STARD3, such that when ER contact with endocytic organelles is reduced, resulting in cholesterol accumulation, STARD3 instead promotes association between the cholesterol-laden lysosomes and mitochondria.

Taken together, our data demonstrate a role for NPC1 in tethering ER contact sites with late endocytic organelles that mediate cholesterol transport to the ER. In NPC1-deficient cells, where ER contact is reduced, cholesterol accumulates in lysosomes, which form increased contact with mitochondria, likely underlying the mitochondrial cholesterol accumulation and dysfunction in NPC.

## Discussion

In this study we have demonstrated an important function for MCS between the ER and endocytic organelles in transport of LDL-cholesterol from endocytic organelles to the ER. MCS regulate multiple mechanisms that could contribute to endocytic cholesterol export. ER-endosome contacts define sites of endosome fission^29^, possibly of lipid-rich vesicles, for recycling to the plasma membrane and MCS are also required for retrograde transport from endosomes to the Golgi^30^, another proposed route of cholesterol transport^31^. The majority of LDL-derived cholesterol is thought to be trafficked to the ER via the plasma membrane, to safeguard supply. However, approximately 30% of total LDL-cholesterol is estimated to undergo direct transport from the endocytic pathway to the ER for esterification^6^, by an as yet unclear mechanism. Here we present several lines of evidence for direct transport of LDL-cholesterol from endocytic organelles to the ER across MCS between the two organelles.

Firstly, we uncovered a role for NPC1 as a functional MCS tethering protein that satisfies all the major criteria of a tether^32^: it is enriched at MCS where it interacts with ER-localised Gramd1b and is functional in mediating MCS formation. Gramd1 proteins are known to transfer sterols via their StART-like domain and Gramd1b efficiently transports cholesterol^33^ and has been implicated in transport of HDL-derived cholesterol to the ER at the ER-plasma membrane interface^23^. Gramd1b recruitment to ER-plasma MCS was dependent on cholesterol-loading of the plasma membrane. We found that Gramd1b also interacts with NPC1 at ER-lysosome contact sites when endosomal cholesterol is replete, suggesting that this sterol transfer protein is targeted to contact sites with cholesterol-rich organelles. Our data support a model of Gramd1b recruitment to lysosomes under conditions of high LDL-cholesterol in the endocytic pathway, where it interacts with NPC1 to stabilise the interorganellar association for transport of cholesterol to the ER. However, the accumulation of unesterified, filipin-stained cholesterol in endocytic organelles was more pronounced in the absence of NPC1 than Gramd1b, even though uptake of fluorescent-LDL was unaffected. This indicates a dual role for NPC1, both in direct transport at Gramd1b-dependent contact sites and in indirect transport via the plasma membrane^34^. Indeed NPC1 was previously shown to mediate Rab8-dependent recycling of LDL-derived cholesterol to the plasma membrane^34^. Given the comparable reduction of cholesterol esterification in NPC1 or Gramd1b-depleted cells, Gramd1b is likely to also play a dual role in cholesterol transport by contributing both to indirect transport via the plasma membrane^23^ as well as via ER-lysosome contact sites. Interestingly, Gramd1b has the unique ability to also transfer phosphoinositides^33^, suggesting that it may function in a phosphoinositide-sterol counter exchange mechanism as has been described for OSBP at the ER-Golgi interface^35^.

In addition to measuring cholesterol both with filipin staining and using the Amplex Red assay, we have also demonstrated the use of a “click”-cholesterol probe to image cholesterol transport. The probe, complexed with BSA for entry into the endocytic pathway, was redistributed from the lysosome to the ER after a one hour chase in wildtype but not NPC1-deficient cells, consistent with the well documented defect in cholesterol egress in NPC. It is, however, possible that a proportion of the probe was trafficked from the plasma membrane to the ER in control cells rather than across lysosome-ER contact sites. The most compelling evidence for a role for MCS in direct cholesterol transport from the endocytic pathway to the ER, however, is the ability to reverse the cholesterol accumulation in NPC1-deficient cells by artificially tethering late endocytic organelles to the ER. We used a sterol-insensitive ORP1L mutant, that constitutively binds VAP to act as an artificial tether but that cannot transport sterol, to expand ER-lysosome MCS. Remarkably, MCS expansion by overexpression of this artificial tether rescued lysosomal cholesterol accumulation in NPC1-deficient cells. ORP1L was recently shown to mediate cholesterol egress from NPC1-inhibited cells in a PI(4,5)P2/PI(3,4)P2-dependent manner^8^. However, whereas cholesterol transport by ORP1L is dependent on its ORD, here its tethering function alone was sufficient to restore cholesterol egress. Thus even in the absence of NPC1, cholesterol can be transported at lysosome-ER contacts, suggesting that when the contacts are expanded, either another protein, likely endogenous ORP1L or Gramd1b, can compensate for loss of NPC1, or alternatively, cholesterol transport may occur along a concentration gradient across the expanded contact without the need for a sterol transporter. While the precise mechanism of transport remains unclear, that MCS expansion also corrected the reduced esterification phenotype in NPC1-deficient cells indicates that ER-lysosome MCS can act as conduits for the transport of LDL-derived cholesterol to the ER for esterification. Our data thus favours a model of multiple pathways of transport of LDL-cholesterol to the ER^4^, likely operating at different stages of endosome maturation as the LDL cholesterol ester core is progressively hydrolysed. In such a model, a proportion of the LDL cholesterol ester core is only hydrolysed in the more acidic environment of the lysosome that is optimal for acid lipase activity. The resulting remaining LDL-derived cholesterol is cleared from the lysosome across ER-lysosome MCS. In the absence of NPC1, two important pathways for cholesterol transport to the ER are disrupted: Rab8-dependent cholesterol recycling to the plasma membrane and direct transport across ER-lysosome MCS. However, the cholesterol that accumulates in the lysosome as a consequence of NPC1 loss can be directly transported to the ER by artificial expansion of the ER-lysosome interface.

It is interesting that cholesterol egress can be restored even in the absence of NPC1, which has been shown to receive cholesterol from luminal NPC2^36^. NPC2 can transfer cholesterol between phospholipid liposomes in the absence of NPC1^37^ and is thought to transfer cholesterol directly to the limiting membrane, as well as to other transmembrane proteins^38^. NPC2 was also shown to contribute to endosomal cholesterol transport to the mitochondria in an NPC1-independent manner^39^. Thus, in cells lacking NPC2, even though NPC1/Gramd1b-mediated contacts are still formed, cholesterol is no longer delivered to the limiting membrane of late endosomes/lysosomes and is therefore inaccessible for transport across the contact. The restoration of cholesterol egress by MCS expansion when NPC1 is inhibited may offer potential for the development of novel therapeutics for NPC and other lysosomal storage diseases.

**Fig. 7:**
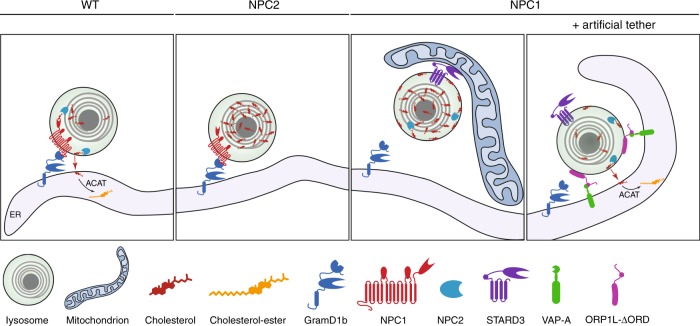
Reciprocal relationship between lysosomal cholesterol and NPC1-regulated MCS. A schematic representation of cholesterol transport at NPC1-regulated lysosomal MCSs. In the presence of cholesterol in the endocytic pathway, lysosomal NPC1 interacts with Gramd1b on the ER to tether MCSs that mediate the transport of cholesterol from the endosome to the ER for esterification by ACAT. In the absence of functional NPC2, luminal cholesterol fails to be delivered to the limiting membrane for egress at NPC1-Gramd1b tethered MCS and therefore accumulates in the lysosome. In the absence of functional NPC1, NPC1-tethered lysosome-ER contact sites are lost and cholesterol accumulates in the lysosome. Under these conditions STARD3 mediates extended lysosome contact sites with mitochondria. However, when the lysosome is artificially tethered to the ER by expression of an ORP1L mutant with its sterol-binding domain deleted (ORP1L-ΔORD), cholesterol transport to the ER is restored

Imaging MCS by EM, we have uncovered an unexpected expansion of an MCS population that forms between lysosomes and mitochondria in cells depleted of NPC1 or Gramd1b. Vacuole-mitochondria MCS were previously identified in yeast^40,41^, where the extent of different MCS populations is coordinated by enrichment of the sterol-binding protein Lam6 at different MCS populations in response to the physiological condition^42^. Similarly we found the localisation of the sterol-binding protein, STARD3, at lysosome MCS with the ER versus mitochondria to be dependent on the sterol environment of the endocytic pathway. In mammalian cells, lysosome-mitochondria contacts have been described in developing erythroid cells^43^, and in hypoxic cells, likely functioning in lysosome-mediated degradation of damaged outer mitochondrial membrane protein^44^. Four sub-populations of lysosome-mitochondria MCS were recently described^45^ and reciprocal regulation of lysosomal Rab7 activity and mitochondrial dynamics has been demonstrated at these MCS^46^. Previous studies have shown that cholesterol accumulation in NPC cells inhibits Rab7 GTP hydrolysis, resulting in elevated membrane-associated Rab7^47^. Interestingly, Rab7 hydrolysis at the lysosome-mitochondria interface is required for MCS disassembly^46^. Thus, the resistance of Rab7 GTP to hydrolysis in cholesterol-laden lysosomes might contribute to the extended mitochondrial MCS in NPC1 or Gramd1b-deficient cells. This expansion of lysosome-mitochondria MCS may offer an alternative route to clear cholesterol from the endocytic pathway and a potential mechanism for the elevated cholesterol in the mitochondria of NPC1-deficient cells^26^. The observation that STARD3 depletion reduced lysosome-mitochondria contacts to a level below that in control cells indicates an essential role for STARD3 in the formation of this MCS population. In the context of STARD3’s known role in regulating cholesterol transport to the mitochondria^26^, our data suggest a function for lysosome-mitochondria MCS in cholesterol transport, possibly in an attempt to rescue cholesterol-induced lysosomal stress. However, the resulting cholesterol accumulation in the mitochondria is associated with mitochondrial dysfunction in NPC^48,49^. Indeed increased mitochondrial cholesterol content on STARD3 overexpression resulted in reduced ATP generation, increased mitochondrial superoxide levels and other indicators of mitochondrial dysfunction^50^. The absence of expanded ER-endosome MCS in STARD3-overexpressing cells when treated with U18666A, where endocytic organelles are laden with lipid, suggests that like ORP1L, STARD3 interaction with VAP may also be sterol sensitive. Thus, a picture of sterol responsive late endosome/lysosome MCS populations is emerging (summarised in Fig. 7) whereby in the presence of sterols, NPC1 interacts with Gramd1b to provide platforms for LDL-derived cholesterol transport to the ER that is dependent on luminal NPC2 to deliver the cholesterol to the limiting membrane. When this fails and cholesterol accumulates in endocytic organelles, STARD3 mediates expansion of lysosome-mitochondria contacts, though how it anchors to the mitochondria is as yet undetermined. Critically, expansion of the ER-lysosome interface corrects the cholesterol accumulation in NPC1-deficient cells.

Taken together, our data demonstrate an essential role for MCS in LDL-cholesterol egress and describe a role for NPC1 in establishing contacts between late endosomes/lysosomes and the ER, thereby challenging the notion that the sole function of NPC1 is that of a cholesterol transport protein. Our findings instead indicate that, through the interaction with Gramd1b, NPC1 also plays an important role in the formation of MCS, which serve as conduits for cholesterol transport. In NPC1-deficient cells, where cholesterol transport to the ER is impaired, STARD3 promotes the association of cholesterol-laden lysosomes with mitochondria, providing a likely mechanism for the mitochondrial cholesterol accumulation and dysfunction in NPC. These data, together with the sterol-dependent interaction between NPC1 and Gramd1b, demonstrate how heavily influenced late endocytic organelle MCS populations are by the sterol environment. Thus sterol egress from the endocytic pathway at MCS is regulated, at least in part, by the cholesterol to be transported, with NPC1-tethered MCS playing a central and sterol-responsive role in regulating the sterol environment of the endocytic pathway.

## Methods

### Cell culture and transfection

HeLa (ATCC: CCL2), CHO (WT: ATCC CRL-9618, NPC: CHO-M12, PMID 10964915) and fibroblast (Coriell Institute: GM05399 Control, GM03123/GM22870 NPC1 patient, GM18455 NPC2 patient) cells were cultured in DMEM/10% FBS (Invitrogen). Cells were transfected using Lipofectamine LTX Plus for plasmids and Lipofectamine RNAiMax (Invitrogen) for siRNAs, according to manufacturer’s instructions. HeLa NPC1^−/−^ cells in which NPC1 was knocked out using CRISPR/Cas9 genome editing were kindly provided by Dr. Wim Annaert (VIB Center for Brain & Disease Research, Leuven, Belgium). (PMID: 28134274).

### Antibodies

Mouse anti-VAPA (MAB5820) and VAPB (MAB58551) antibodies were from R&D Systems (diluted 1:250 and 1:500 respectively), rabbit anti-LAMP1 was purchased from Cell Signalling (D2D11, diluted 1:100), rabbit anti-NPC1 (immunoEM, diluted 1:50) was a kind gift from Elina Ikonen (University of Helisinki), rabbit anti-NPC1 (WB, diluted 1:2000) and rabbit anti-Gramd1b (diluted 1:800 for WB, 1:10 for immunno-EM) were from Abcam (EPR5209 and ab154934 respectively), and rabbit anti-STARD3 (diluted 1:1000) was a kind gift from Catherine Tomasetto (IGBMC, France). Antibody to the extracellular domain of EGFR used for gold conjugation was isolated from the Mab 108 hybridoma (ATCC, HB-9764).

### Plasmids

wtORP1L-GFP, ORP1L-deltaORD-GFP and ORP1L-FFATmut-GFP were a kind gift from Jacques Neefjes (Netherlands Cancer Institute). STARD3-GFP was a kind gift from Fabien Alpy (IGBMC, France). Gramd1-GFP plasmids were a kind gift from Alberto Gatta/Tim Levine (UCL). To generate NPC1-GFP, NPC1 was cloned from pSG5-NPC1 (kind gift from Yiannis Ioannou, Mount Sinai School of Medicine) into pEGFP-C1 at EcoR1/BamH1 sites. APEX2-GBP was a gift from Rob Parton (Addgene plasmid #67651). Sec61-GFP plasmid was a gift from Eric Schirmer (Addgene plasmid #26008).

### siRNAs

The non-targeting control siRNA was Allstars negative control siRNA (Qiagen 1027281). siRNA smart pools targeting VAPA (M-021382-01), VAPB (M-017795-00) and Gramd1b (M-026529-01) were synthesized by Dharmacon. siRNA targeting NPC1^15^ (target sequence: 5′-ACCAATTGTGATAGCAATATT-3′), and STARD3 (target sequence: 5′-AACACAGGCATCCGTAAGAAC-3′) were synthesized by Qiagen.

### Western blotting and immunoprecipitation

For Western blotting, cells were lysed in lysis buffer [40 mM HEPES, 80 mM NaCl, 10 mM EDTA, 10 mM EGTA, 1% Triton-X100, protease inhibitor cocktail (Calbiochem set I), phosphatase inhibitor cocktail (Calbiochem set II)]. Lysates were fractioned by SDS–PAGE on 10% gels under reducing conditions and immunoblotted on nitrocellulose membranes. Following incubation with infrared-fluorophore-conjugated secondary antibodies (1:8000), membranes were scanned in an Odyssey SA scanner (LI-COR Biosciences). For IP, cells were lysed as above following crosslinking for 30 min at RT in DSP crosslinking solution (1 mM DSP, 10 mM Triethanolamine, 0.25 M sucrose, 2 mM CaCl2, pH 7.4) and blocking for 30 min at RT in blocking solution (10 mM triethanolamine, 0.25 M sucrose, 2 mM CaCl2, 50 mM Ethanolamine, pH 7.4). NPC1 immunoprecipitates were fractioned, immunoblotted and scanned as described for Western blotting. Supplementary Fig. 7 shows uncropped blots for Fig. 2a, c.

### Electron microscopy

For conventional EM, cells were serum starved for 1 h prior to stimulation with 100 ng/ml EGF (Sigma) with 10 nm anti-EGFR gold conjugate in DMEM/0.2% BSA. After fixation in 2% paraformaldehyde (PFA)/2% glutaraldehyde for 30 min, cells were post-fixed in 1% osmium tetroxide, 1.5% potassium ferricyanide, incubated in 1% uranyl acetate, dehydrated and embedded in TAAB-812 resin. For U18666A-mediated inhibition of NPC1, cells were incubated with 2 µg/mL U18666A (Calbiochem) for 18 h prior to fixation. Cells transfected with APEX2-GBP were incubated with 1.5 mg/ml DAB (TAAB) in TRIS buffer supplemented with 0.02% H_2_O_2_ for 30 min following fixation. For correlative light and electron microscopy (CLEM), live cell imaging of cells expressing NPC1-GFP cultured on gridded dishes (Mat-tek) was followed by fixation and embedding for EM. For Filipin staining, cells were fixed in 4% PFA prior to incubation with 100 μγ/μλ Filipin (Sigma) in PBS for 45 min and imaged by fluorescent microscopy prior to fixation and embedding for EM. Expressing cells were identified using grid coordinates and imaged by EM. Electron micrographs were montaged in Adobe Photoshop and fluorescent images overlaid.

### Immuno-EM

For pre-embedding labelling, cells were fixed in 4% PFA, permeabilised in either 40 μg/ml digitonin (Calbiochem) in PBS for 15 min for NPC1 or Gramd1b labelling, or 0.05% triton in PHEM (60 mM Pipes, 25 mM Hepes, 10 mM EGTA, 2 mM MgCl_2_, PH 7) for 5 min on ice for PFO labelling and incubated with anti-NPC1 (1:25) or PFO-gst (20 μg/ml) followed by rabbit anti-GST (1:40) and nanogold-secondary antibodies (Nanoprobes, 1:200) prior to fixation for EM in 2% PFA/2% glutaraldehyde, followed by gold enhancement (Nanoprobes), according to manufacturer’s instructions. Post-fixation and embedding were as for conventional EM. For cryo-immunoEM, cells were fixed in 4% PFA/0.1% Gluteraldehyde in 0.1 M phosphate buffer for 2 h at RT, scraped in 1% gelatin and pellets resuspended in 12% gelatin. Pelleted cells were cooled on ice and 1 mm square blocks were cut and infused with 2.3 M sucrose rotating at 4 °C overnight. Blocks were mounted onto pins in liquid nitrogen and 80 nm sections cut at −120 °C. Ultrathin cryo-sections were collected in 2% methyl cellulose/2.3 M sucrose onto formvar-coated copper grids prior to labelling with 20 μg/ml PFO-GST^25^ followed by rabbit anti-GST (1:40) and proteinA-gold (1:50). Sections were stained with 1.8% methyl cellulose/0.4% uranyl acetate and imaged by EM.

### EM quantitation

EGFR-MVBs were distinguished from nonEGFR-MVBs by the presence of gold particles and lysosomes were identified by the presence of multi-lamellar whorls. MCS between the ER and MVBs were classified as areas where the opposing membrane were within 30 nm of each other with no minimum length. For quantitation of %endocytic organelle membrane in contact with the ER, the sum of MCS lengths (measured in ImageJ)/organelle was divided by the total length of the organelle limiting membrane. A minimum of 60 organelles was included for each condition. For MCS quantitation, means, standard errors of means (SEM) and paired, two-tailed students *T*-tests were calculated in excel, with *p* values > 0.05 represented as not significant (ns), * < 0.05, ** < 0.01 and *** < 0.001.

### Fluorescence imaging

For live-cell imaging, cells were cultured in an 8-well glass bottom slide (Ibidi, #80827) and transfected with Sec61-GFP DNA (75 ng per well). Immediately prior to imaging, cells were labelled with 100 nM Lysotracker Red DND-99 (Thermo Fisher, #L7528) for 10 min and washed with imaging buffer (20 mM HEPES, 115 mM NaCl, 1.2 mM MgCl_2_, 1.2 mM K_2_HPO_4_, 1.8 mM CaCl_2_ and 0.2 % (w/v) glucose). Cells were kept in imaging buffer for the duration of image acquisition on a Leica TCS SP8 microscocpe.

For imaging of fixed cells, cells were cultured onto 11 mm coverslips placed in wells of a 24-well plate and transfected with the plasmids of interest (ORP1L-GFP, ORP1L-ΔORD-GFP, ORP1L-FFATmut-GFP). The medium was replaced with DMEM for 1 h and cells were fixed with 4% PFA for 1 h at room temperature. After quenching of the paraformaldehyde with glycine (1.5 mg/ml PBS) for 10 min, cells were stained with Filipin (0.05 mg/ml in PBS/10%FBS) for 2 h at room temperature. The coverslips were washed, mounted onto glass slides using Prolong Gold antifade reagent (Invitrogen) and imaged on a Leica TCS SP8 microscope and LAS X software. For CLEM, cells were imaged with a Nikon Eclipse Ti-E. For LDL uptake, cells were incubated with 25 μγ/μλ DiI-LDL (Invitrogen) for 10 min, washed and chased in full medium for 10 min prior to fixation with 4% PFA in PBS for 20 min. Images were acquired with a Leica TCS SP8 microscope and LAS X software.

### Visualization of clickable and photocrosslinkable cholesterol (pacChol) in cells

Cells were seeded onto 11 mm coverslips placed in wells of a 24-well plate to 65–75% confluency PacChol/BSA complexes were prepared according to the protocol of Martínez et al.^51^. Brielfy, 0.6 mg pacChol was dissolved in 60 µl EtOH (1 % (w/v)) and diluted with 60 µl H_2_O. The pacChol, EtOH and H_2_O mixture was vortexed and centrifuged (2000 rpm, 10 min, 4 °C). The supernatant was discarded, and the pellet was resuspended in 60 µl 0.25 M sucrose/1 mM EDTA, pH 7.3. 24 mg fatty acid-free BSA were added and dissolved while shaking at RT. Once the BSA was completely dissolved, the pacChol/BSA mixture was centrifuged (12000 rpm, 10 min, 4 °C). The supernatant containing pacChol/BSA was collected and used for cell labelling. Cells were starved for 2 h in serum-free DMEM, labelled with 20 µM pacChol/BSA complex for 20 min and chased for indicated times in serum-free DMEM. Subsequently, cells were washed with 1 mL PBS/0.01 mM CD for 5 min at 37 °C followed by two washes with PBS at RT. Cells were overlaid with 0.5 ml of cold imaging buffer (20 mM HEPES, 115 mM NaCL, 1.2 mM MgCl_2_, 1.2 mM glucose and 1.8 mM CaCl_2_, pH 7.4/NaOH), and UV-irradiated (λ~365 nm) on ice for 5 min. Cells were immediately fixed with pre-cooled MeOH at −20 °C for 20 min. Non–cross-linked lipids were extracted by washing three times with 0.78 mL of CHCl_3_/MeOH/AcOH (10:55:0.75) (v/v) and twice with PBS. Cells were then incubated with 50 μl of click mixture (1 µl 2 mM Alexa-488-azide or Alexa-555-azide, 125 µl 10 mM Cu(I)BF_4_ in acetonitril and 0.5 ml PBS) for 1 h at room temperature in the dark. Cells were then washed with PBS and incubated with 50 μL of primary α-LAMP1 antibody (1:200 in PBS supplemented with 2% BSA and 0.3% Triton X-100) for 1 h in the dark. Coverslips were briefly washed with PBS and incubated with secondary antibody (α-rabbit conjugated to AlexaFluor488, 1:800) for 30 min–1 h, washed briefly with PBS, and mounted in ProLong Gold Antifade mounting medium. Microscopy images were captured using a confocal laser scanning microscope (Zeiss LSM800) with a 63× oil objective.

### Image analysis

Images were analysed on Fiji (W. Rasband, NIH, USA) using the FluoQ macro^52^ for automatic extraction of intensity and co-localization values. These values were subsequently loaded in R and grouped according to conditions. Graphs were generated using the ggplot2 package in R^53^.

### Measurement of cholesterol and cholesterol esters

For the Amplex Red Cholesterol Assay (Thermo Fisher Scientific, CA, USA), aliquots of cell pellet sonicates were incubated for 30 min at 37 °C in the presence of 2 U/mL of HRP, 2 U/mL cholesterol oxidase, and 300 µM Amplex Red reagent. The assay was performed with and without addition of 0.2 U/mL cholesterol esterase, and esters determined by calculating the total minus free cholesterol. Fluorescence was measured with an Infinite M1000 pro plate reader (Tecan Group Ltd., Switzerland).

### Statistical analyses

All biochemical data were from biological triplicates unless otherwise indicated, whereas imaging data was quantified from multiple cells as indicated in the figure legends. Statistical analysis was performed with a Welch two sample t-test for comparison of two groups using R. Significance is indicated using asterisks (***P ≤ 0.001, **P ≤ 0.01, *P ≤ 0.05)

## Supplementary information

Supplementary Information

Description of Additional Supplementary Files

Supplementary Movie 1

## Data Availability

Data supporting the findings in this study are available from the corresponding author upon reasonable request.
