# Supplementary material for: NPC1 regulates ER contacts with endocytic organelles to mediate cholesterol egress
Source: Nat Commun. 2019 Sep 19;10:4276. doi: 10.1038/s41467-019-12152-2 (PMC6753064; doi:10.1038/s41467-019-12152-2)
Supplement: Supplementary file 1 — Supplementary Information [file 41467_2019_12152_MOESM1_ESM.pdf]

Supplementary Information

NPC1 regulates ER contacts with endocytic organelles to mediate cholesterol egress.

Höglinger et al.

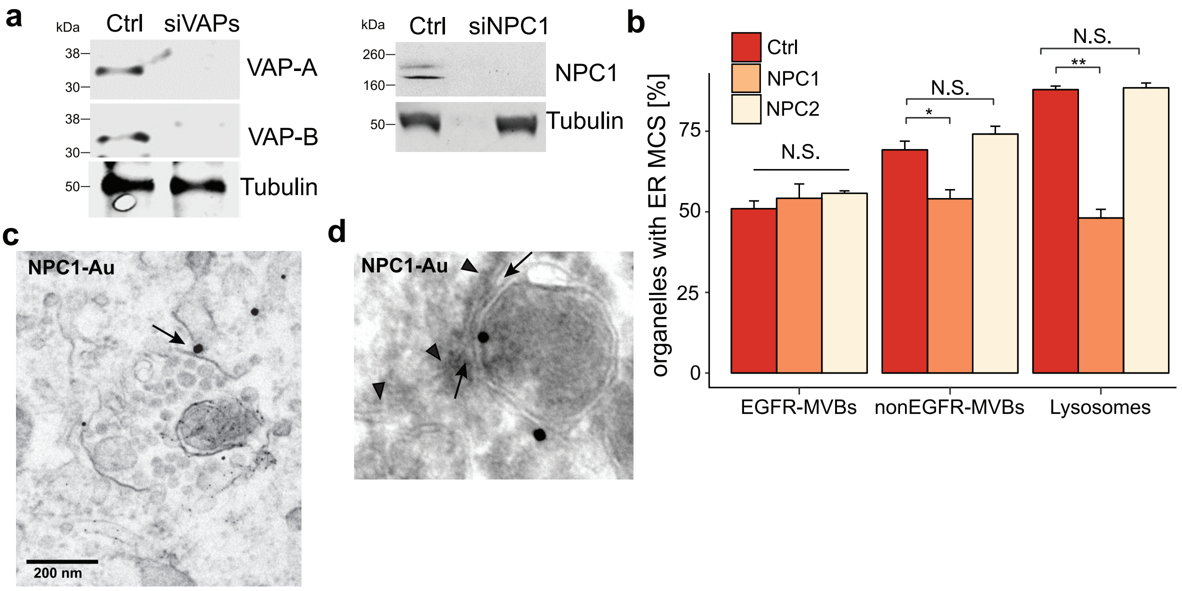

Supplementary Figure 1: NPC1 regulates MCS formation. a) Whole cell lysates from HeLa cells treated with control siRNA (Ctrl) or siRNA targeting VAP-A and VAP-B (siVAPs) or NPC1 (siNPC1) were blotted with antibodies as indicated. **b)** Endocytic organelle populations in in control fibroblasts as well as in NPC1 and NPC2 patient fibroblasts were scored according to the presence of MCS with the ER and the percentage of organelles with an ER MCS quantified. Data shown is the mean of three independent experiments + *sem*. Welch two sample t-tests were performed between control and patient lines for each vesicle population (EGFR-MVBs: N.S. *P*=0.568, *P*=0.179, nonEGFR-MVBs: */N.S. *P*=0.0176, *P*=0.249, Lysosomes: **/N.S., *P*=0.00153, *P*=0.789) **c)** and **d**) Representative electron micrographs showing NPC1 staining at late endocytic organelle contact sites with the ER using pre-embedding labeling (c) or cryo-immunoEM (d). Arrowheads, ER. Arrows, MCS.

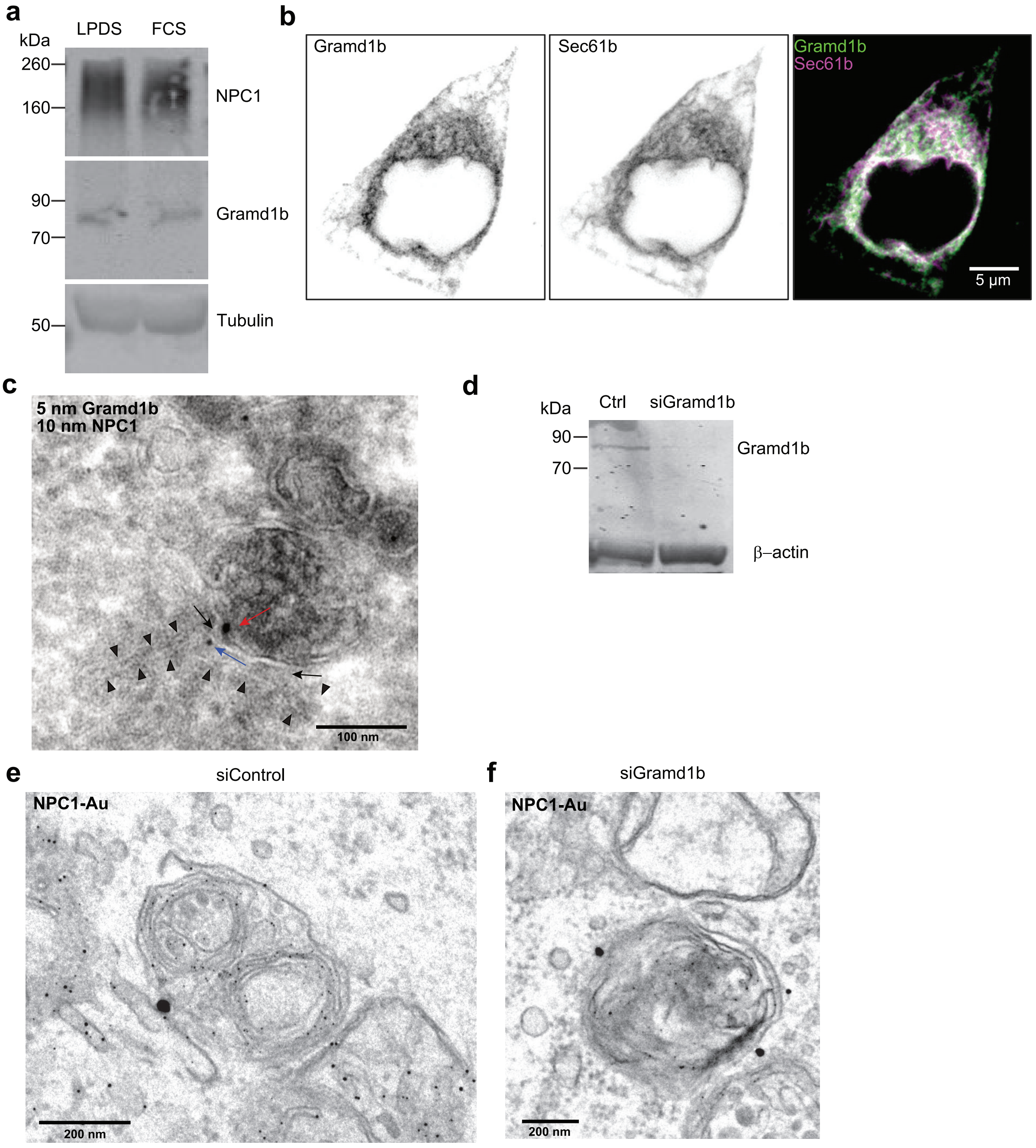

Supplementary Figure 2: NPC1 interacts with the ER-localised sterol transport protein Gramd1b at MCSs. a) Cell lysates from HeLa cells cultured in medium containing 10% FCS or LPDS were blotted with antibodies as indicated. **b)** Live-cell confocal microscopy images of HeLa cells transfected with Gramd1b-GFP and mCherry-Sec61b. Scale bar, 5 µm. **c)** Cryo-immunoEM of endogenous NPC1 (10 nm, red arrow) and Gramd1b (5nm, blue arrow) at ER-endosome MCSs (black arrows) in HeLa cells. Arrowheads indicate ER. Scale bar, 100 nm. **d)** Cell lysates from HeLa cells treated with control siRNA (Ctrl) or siRNA targeting Gramd1b were blotted with antibodies as indicated. **e) and f)** Representative electron micrographs showing endogenous NPC1 staining at an ER-endolysosome MCS in control HeLa cells (e) but not in HeLa cells depleted of Gramd1b (f). Scale bar, 200 nm.

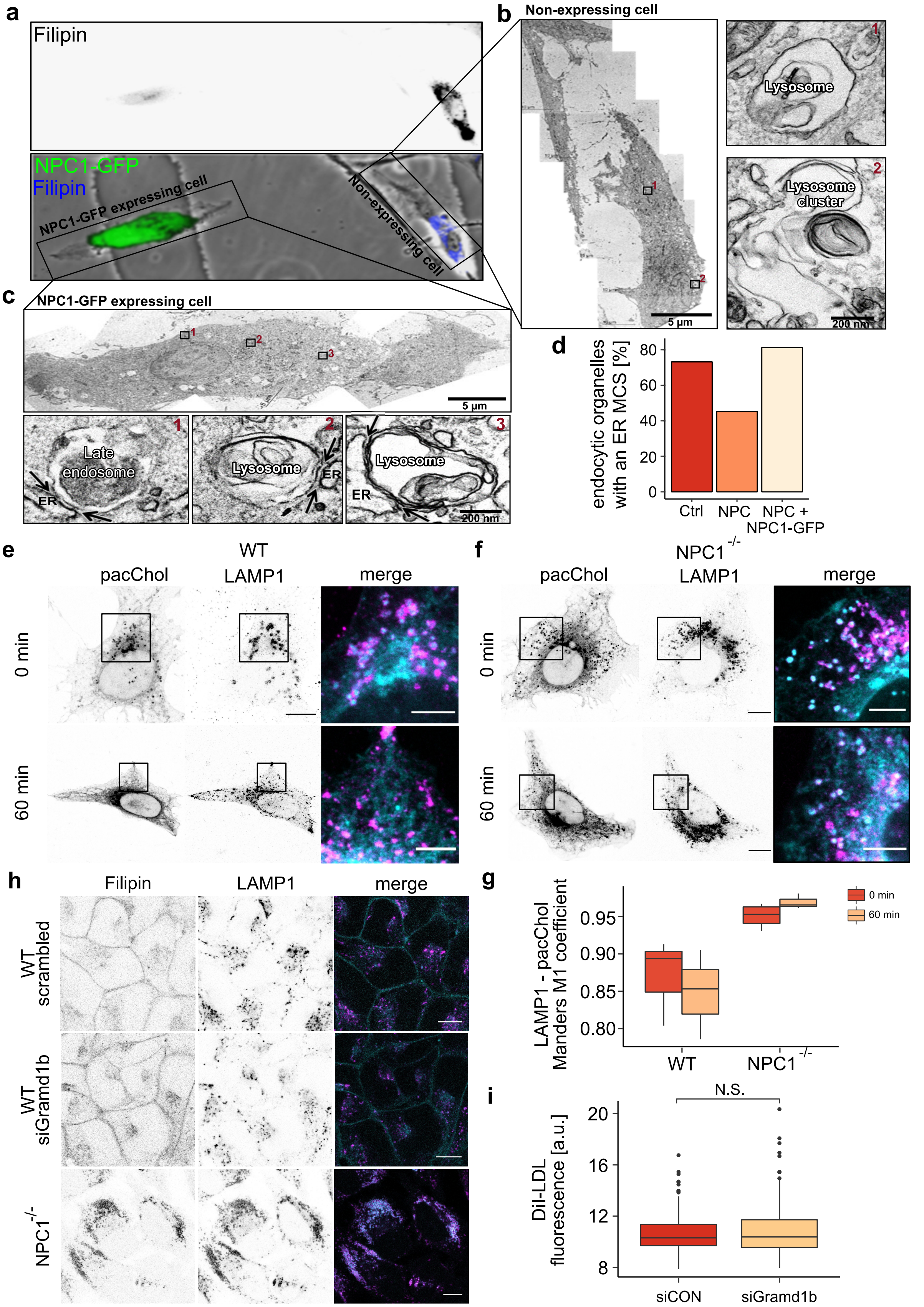

Supplementary Figure 3: NPC1-regulated MCSs mediate cholesterol transport from late endocytic organelles to the ER. a) NPC-patient fibroblasts cultured on gridded dishes were

transfected with NPC1-GFP, fixed, filipin-stained and imaged by light microscopy. **b)** Low magnification electron micrographs of non-expressing cells were montaged (scale bar 5 µm), with boxed regions imaged in high magnification (scale bar 200 nm). In non-expressing cells, MCSs were infrequent (quantified in d)) and lysosomes often appeared clustered and disorganized as shown in panel 2. **c)** NPC1-GFP-expressing cells from a) were located using grid coordinates and imaged by at high magnification (scale bar in boxed region, 200 nm) in order to visualize ER-lysosome MCSs (black arrows). **d)** The percentage of endocytic organelles with an ER contact site was quantified by EM in control (Ctrl) or NPC-patient fibroblasts (NPC) or NPC-patient fibroblasts expressing NPC1-GFP (NPC+NPC1-GFP). **e)** and **f)** Confocal microscopy images of cholesterol localization in HeLa cells. WT (e) and $NPC1^{-/-}$ (f) cells were pulsed with photoactivatable and clickable cholesterol (pacChol) complexed to fatty-acid free bovine serum albumin for 20 min and chased in serum-free medium for the indicated times. pacChol was UV-crosslinked, cells were fixed, non-crosslinked lipids were washed away and crosslinked lipids were stained by click-reaction with fluorophores (cyan in merged image). Late endosomes and lysosomes were identified using anti-LAMP1 antibody (magenta in merged image). Enlargements of boxed areas are shown in the merged images. Scale bars, 10 µm. **g)** Quantification of cholesterol and lysosomal co-localization. The Manders M1 coefficient between pacChol and LAMP1 channels was extracted for each cell (WT/0 min: n=3 cells, WT/60 min: n=3 cells, $NPC1^{-/-}$/0 min: n=4 cells, $NPC1^{-/-}$/60 min: n=3 cells) and presented as boxplots. **h)** Confocal microscopy images of WT HeLa cells transfected with control (scrambled) or Gramd1b siRNA as well as $NPC1^{-/-}$ HeLa cells stained with 50 µg/mL Filipin. Late endosomes and lysosomes are visualized by immunofluorescence using anti-LAMP1 antibody. Scale bars represent 10 µm. **i)** DiI-LDL fluorescence intensities of HeLa cells transfected with either control siRNA (siCON, n=231 cells) or siRNA targeting Gramd1b (siGramd1b, n=200 cells). Centre lines show medians, box limits indicate first (Q1) and third quartiles (Q3), whiskers extend to a maximum distance of 1.5*IQR (interquartile range) from Q1 and Q3, respectively or to the most extreme datapoint within that range. Welch two sample t-tests were performed between siCON and siGramd1b conditions (N.S. *P*=0.2476).

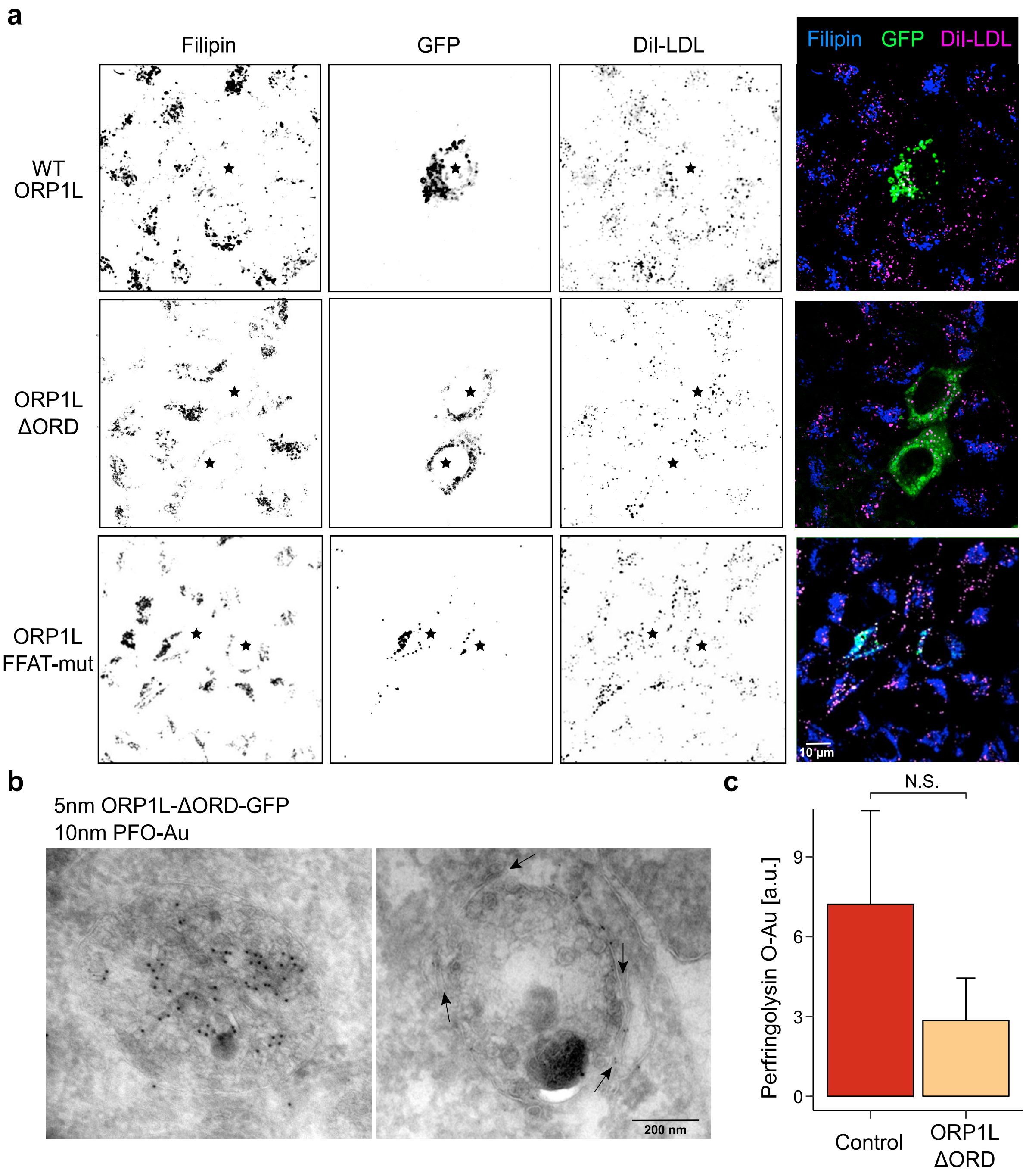

Supplementary Figure 4: Expansion of lysosome-ER contact sites does not affect LDL uptake but restores cholesterol egress in NPC1-deficient cells. a) HeLa cells transfected with wtORP1L, ORP1L-ΔORD or ORP1L-FFATmutant-GFP constructs were treated with U18666A (2 µg/mL for 18h) prior to a 10 min pulse with DiI-LDL, followed by a 10 min chase in full medium. Cells were fixed, stained with filipin and imaged by light microscopy. Scale bar, 10 µm. **b)** HeLa cells transfected with ORP1L-ΔORD-GFP were treated with U18666A (2 µg/mL for 18h). Ultrathin cryo sections were labeled with PFO/10 nm-Au and anti-GFP/5 nm-Au. Scale bar, 200 nm. **c)** The number of PFO-Au per endocytic organelle was quantified in cells not expressing (Control) or expressing ORP1L-ΔORD-GFP. Data are presented as mean of two independent experiments + *sem*. Welch two sample t-tests were performed between Ctrl and ORP1L-ΔORD conditions (N.S. P=0.2723).

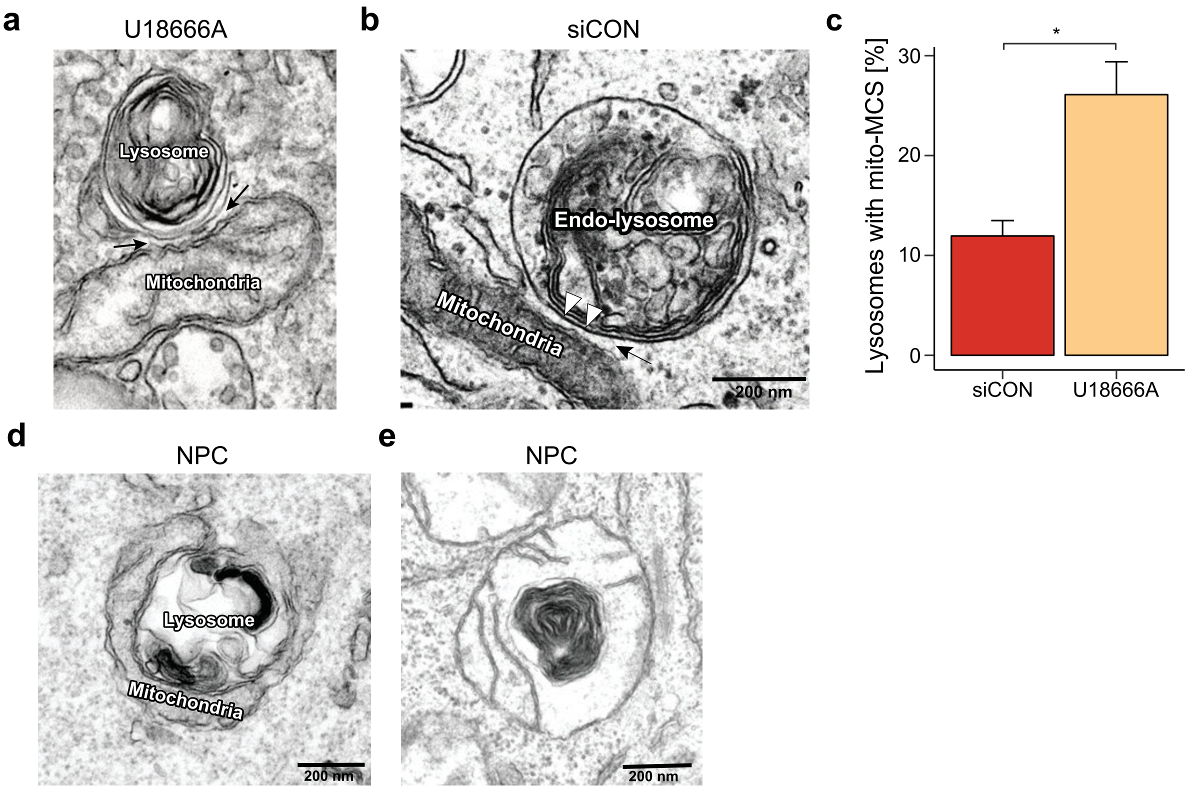

Supplementary Figure 5: Increased lysosome-mitochondria MCSs in NPC1-deficient cells. a) Electron micrograph showing a lysosome-mitochondria MCS (black arrow) in HeLa cells treated with U18666A (2 µg/mL for 18h. **b)** Electron micrograph showing a lysosome-mitochondria MCS (black arrow) in HeLa cells treated with control siRNA (siCON). White arrowheads indicate tethers visible between the organelles. **c)** The percentage of endocytic organelles with a mitochondria MCS from a) and b) were quantified and expressed as the mean of three independent experiments + *sem*. Welch two sample t-tests were performed between siCON and U18666A conditions (* P=0.03308) **d)** Electron micrograph showing a mitochondrion surrounding a lysosome in NPC-patient fibroblasts. **e)** Electron micrograph showing apparent engulfment of a lysosome by a mitochondrion in NPC-patient fibroblasts. Scale bar, 200 nm.

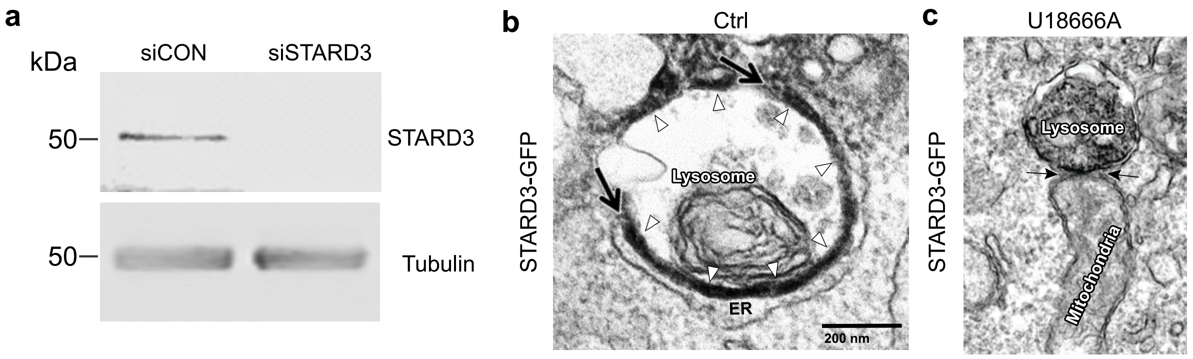

Supplementary Figure 6: STARD3-dependent association of mitochondria with cholesterol-rich lysosomes with reduced ER contact. a) HeLa cells transfected with a non-targeting control siRNA (siCON), or siRNA targeting STARD3 were blotted with antibodies as indicated. **b)** STARD3-GFP was co-expressed with APEX2-GBP in HeLa cells prior to preparation for EM. STARD3-GFP, visible as an electron dense APEX reaction product (white arrowheads) localizes to extended MCSs (black arrows) between late endocytic organelles and the ER. Scale bar, 200 nm. **c)** STARD3-GFP was co-expressed with APEX2-GBP in HeLa cells treated with U18666A (2 μg/mL for 18h) prior to preparation for EM. When NPC1 is inhibited, STARD3-GFP localizes to MCSs (black arrows) between lysosomes and the mitochondria. Scale bar, 200 nm.

Figure 2a blot

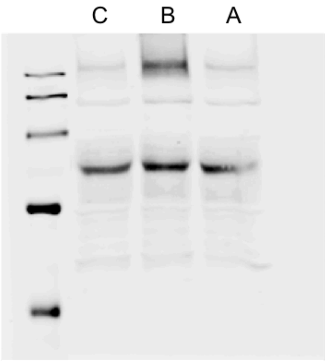

Figure 2c blot

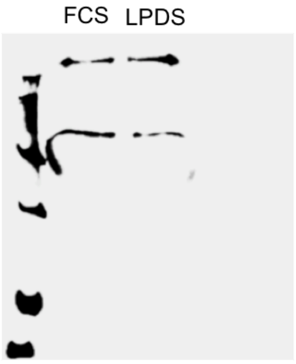

Supplementary Figure 7: Blots from Figure 2.
