# Supplementary material for: NPC1 regulates ER contacts with endocytic organelles to mediate cholesterol egress
Source: Nat Commun. 2019 Sep 19;10:4276. doi: 10.1038/s41467-019-12152-2 (PMC6753064; doi:10.1038/s41467-019-12152-2)
Supplement: Supplementary file 2 — Description of Additional Supplementary Files [file 41467_2019_12152_MOESM2_ESM.pdf]

Description of Additional Supplementary Files

File name: Supplementary Movie 1
Description: Tomogram of lysosome-mitochondria contact. Tomogram showing a lysosome pushing into, but not fusing with the mitochondrial membrane
